# Progesterone receptor does not improve the performance and test effectiveness of the conventional 3-marker panel, consisting of estrogen receptor, vimentin and carcinoembryonic antigen in distinguishing between primary endocervical and endometrial adenocarcinomas in a tissue microarray extension study

**DOI:** 10.1186/1479-5876-7-37

**Published:** 2009-05-28

**Authors:** Chiung-Ling Liao, Ming-Yung Lee, Yeu-Sheng Tyan, Lai-Fong Kok, Tina S Wu, Chiew-Loon Koo, Po-Hui Wang, Kuan-Chong Chao, Chih-Ping Han

**Affiliations:** 1 Department of Obstetrics and Gynecology, Chung-Shan Medical University Hospital, Taichung, Taiwan; 2 Institute of Medicine, Chung-Shan Medical University, Taichung, Taiwan; 3 Clinical Trial Center, Chung-Shan Medical University Hospital, Taichung, Taiwan; 4 Department of Medical Imaging, Chung-Shan Medical University Hospital, Taichung, Taiwan; 5 Department of Medical Imaging and Radiological Science, Chung-Shan Medical University, Taichung, Taiwan; 6 Department of Pathology, China Medical University Hospital, Taichung, Taiwan; 7 David Geffen School of Medicine, University of California, Los Angeles, Los Angeles, California, USA; 8 Department of Pathology, Chung Shan Medical University Hospital, Taichung, Taiwan; 9 Department of Obstetrics and Gynecology, Taipei Veterans General Hospital, and Division of Obstetrics and Gynecology, Faculty of Medicine, National Yang-Ming University School of Medicine, Taipei, Taiwan

## Abstract

**Objective:**

Endocervical adenocarcinomas (ECA) and endometrial adenocarcinomas (EMA) are uterine malignancies that have differing biological behaviors. The choice of an appropriate therapeutic plan rests on the tumor's site of origin. In this study, we propose to evaluate whether PR adds value to the performance and test effectiveness of the conventional 3-marker (ER/Vim/CEA) panel in distinguishing between primary ECA and EMA.

**Methods:**

A tissue microarray was constructed using paraffin-embedded, formalin-fixed tissues from 38 hysterectomy specimens, including 14 ECA and 24 EMA. Tissue microarray (TMA) sections were immunostained with 4 antibodies, using the avidin-biotin complex (ABC) method for antigen visualization. The staining intensity and extent of the immunohistochemical (IHC) reactions were appraised using a semi-quantitative scoring system.

**Results:**

The three markers (ER, Vim and CEA) and their respective panel expressions showed statistically significant (p < 0.05) frequency differences between ECA and EMA tumors. Although the additional ancillary PR-marker also revealed a significant frequency difference (p < 0.05) between ECA and EMA tumors, it did not demonstrate any supplementary benefit to the 3-marker panel.

**Conclusion:**

According to our data, when histomorphological and clinical doubt exists as to the primary site of origin, we recommend that the conventional 3-marker (ER/Vim/CEA) panel is easier, sufficient and appropriate to use in distinguishing between primary ECA and EMA. Although the 4-marker panel containing PR also reveals statistically significant results, the PR-marker offers no supplemental benefit to the pre-existing 3-marker (ER/Vim/CEA) panel in the diagnostic distinction between ECA and EMA.

## Introduction

From hematoxylin and eosin (H&E) stains, it can be difficult to distinguish between pre-operative or curetting specimens of endocervical adenocarcinomas (ECA) and endometrial adenocarcinomas (EMA). Staging is surgical for EMA; however, for primary ECA, staging is clinical. Treatment protocols may differ substantially between them [1,2].

We have already learned that certain immunohistochemical markers may be helpful in distinguishing between ECA and EMA. McCluggage, et al. (2002) proposed that a panel of immunohistochemical stains, comprised of a 3-marker (ER, CEA and Vim) panel, generally results in a confident preoperative distinction between ECA and EMA[3]. Several other studies have reported that a PR- and p16^INK4a^-marker revealed a significant frequency difference (p < 0.05) between ECA and EMA. However, we were interested to discover whether ancillary PR- or p16^INK4a^-marker testing could produce any supplementary benefit to the traditional 3-marker panel.

Our previous studies have already shown that ancillary p16^INK4a^-marker testing does not add value to the traditional 3-marker (ER, CEA and Vim) or 4-marker (PR, ER, CEA and Vim) panel in distinguishing between these 2 gynecologic malignancies (ECA *vs*. EMA)[4,5]. On the other hand, it is uncertain whether the performance and test effectiveness of the 4-marker (PR, ER, CEA and Vim) panel is superior to the conventional 3-marker (ER, CEA and Vim) panel in distinguishing between ECA and EMA. In order to save on the cost and economize laboratory resources, the purpose of this extension study was to examine whether the additional PR-marker can provide supplemental meaningful value to the conventional 3-marker (ER, CEA and Vim) panel, using IHC on a TMA in tissues from Taiwanese women [6-12].

## Methods

### Study materials

The study materials consisted of slides and selected formalin-fixed, paraffin-embedded tissue blocks from 38 hysterectomy specimens retrieved from the archives of the Tissue Bank of the Clinical Trial Center at Chung-Shan Medical University Hospital. These endocervical (n = 14), and endometrial (n = 24) specimens of known origin were collected between 2004 and 2008. Two board-certified pathologists (CP Han and LF Kok) reviewed all H&E stained slides for these cases. A slide with a representative tumor was selected from each case, and the tumor area of interest was circled. The area corresponding to the selected area on the slide was also circled on the block with an oil marker. All the donors' tissue blocks were sent to the BioChiefdom International Co. Ltd, Taiwan for TMA slide construction. They were cored with a 1.5-mm diameter needle and transferred to a recipient paraffin block. The recipient block was sectioned at 5 μm, and transferred to silanized glass slides.

### Immunohistochemical Staining

Using the avidin-biotin complex (ABC) technique, slides were stained with monoclonal antibodies whose main characteristics are summarized in Table 1. Formalin-fixed, paraffin-embedded tissue array specimens with 1.5-mm, 5 μm individual cores, were deparaffinized in xylene, rehydrated through serial dilutions of alcohol, and washed in PBS (pH 7.2). The pH 7.2 PBS buffer was used for all subsequent washes. Slides were stained with the following monoclonal antibodies: progesterone receptor (PR) (NCL-PGR-312, Leica Microsystems), 1:100 dilution; estrogen receptor (ER) (NCL-L-ER-6F11, Leica Microsystems), 1:100 dilution; vimentin (Vim) (NCL-L-VIM-V9, Leica Microsystems), 1:400 dilution; and carcinoembryonic antigen (CEA) (NCL-L-CEA-2, Leica Microsystems), 1:100 dilution. (Table 1) Negative controls were obtained by excluding the primary antibody. Appropriate positive controls were applied. The slides were mounted for examination and the images captured by the Olympus BX51 microscopic DP71 Digital Camera System for study comparison.

**Table 1:** Antibodies used in this study

Antigen	Clone	Supplier	Dilution	Antigen retrieval
PR	Mouse Monoclonal, 16	Leica Microsystems	1:100	citrate
ER	Mouse Monoclonal, 6F11	Leica Microsystems	1:100	citrate
Vim	Mouse monoclonal, V9	Leica Microsystems	1:400	citrate
CEA	Mouse monoclonal, 12-140-10	Leica Microsystems	1:100	trypsin

### Scoring of immunostaining

In this study, the TMA slides were simultaneously reviewed and scored by the two aforementioned pathologists using a two-headed microscope. Since both nucleic and cytoplasmic IHC scoring algorithms had not been optimized and standardized, all PR, ER, Vim and CEA expressions were interpreted using a German semi-quantitative scoring system for assessing the staining intensity and extent[13]. The intensity of marker expression was quantified using the following scores: 0 = negative, 1 = weakly positive, 2 = moderately positive, 3 = strongly positive. The extent of marker expression was quantified by evaluating the percentage of the positive staining areas in relation to the whole cancer area in the core. A score of 0 points was given for 0% reactivity, 1 point was assigned for 1–10% reactivity, 2 points were assigned for 11–50% reactivity, 3 points were assigned for 51–80% reactivity, and 4 points were assigned for 81–100% reactivity. The final immunoreactive score was determined by multiplying the positive intensity and the positive extent scores, yielding a range from 0 to 12. The threshold for differentiating between a final positive and negative immunostaining was set at 4 for interpretation. This optimal cut-off value, for this study, was determined by using the receiver operating characteristic (ROC) curve analysis (Metz, 1978; Zweig & Campbell, 1993)[14,15]. The results can be expressed by dividing cases into groups with scores of 0 to 3 (essentially negative) and 4 to 12 (at least moderately positive in at least 11–50% of cells). This method of assessment has been widely accepted and used in previous studies [16-20].

### Statistical analysis

A chi-squared test or Fisher exact test was performed to test the frequency difference of immunostaining (positive vs. negative) between each IHC biomarker and the two adenocarcinomas. A score of 0 to 3 was classified as negative, and a score of 4–12 was classified as positive. A Mann-Whitney U-test was used to analyze the immunostaining raw scores between the two adenocarcinomas, given the lack of normally distributed IHC scores. To distinguish between primary ECA and EMA, the sensitivity and specificity of EC-type and EM-type immunoprofiles were compared. Sensitivity was defined as the ratio of accurate expression of a typical immunoprofile type among the primary adenocarcinoma of origin. Specificity was defined as the number of tissues that lack typical immunoprofile type expression over the number of tissues that were not actually the primary adenocarcinoma of origin[21]. All analyses were performed using SPSS statistical software (SPSS, Inc., Chicago, IL). All tests were 2-sided and the significance level was 0.05.

## Results

**Figure 1:**
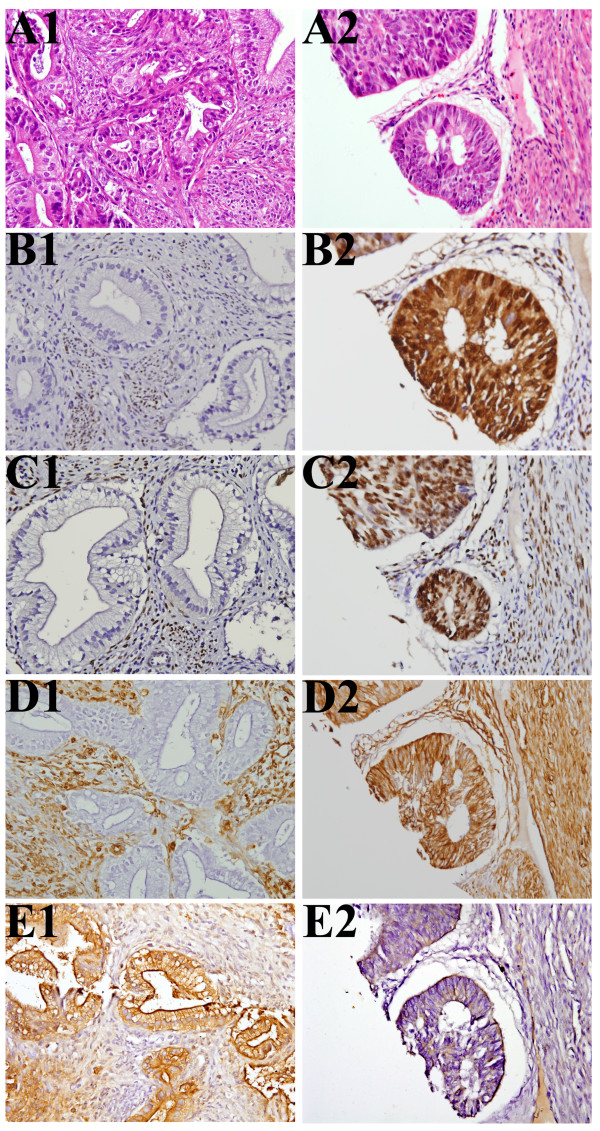
**H&E and immunohistochemical stains for PR, ER, Vim and CEA identified in endocervical adenocarcinomas (ECA) versus endometrial adenocarcinomas (EMA)**. A1, B1, C1, D1 and E1 came from one case with ECA, while A2, B2, C2, D2 and E2 came from another case with EMA. (A1) Adenocarcinoma of endocervix, endocervical type, H&E stain. (A2) Adenocarcinoma of endometrium, endometroid type, H&E stain. (B1) Progesterone receptor (PR) IHC score of ECA tumor cells was 0. (B2) Progesterone receptor (PR) IHC score of EMA tumor cells was 12. (C1) Estrogen receptor (PR) IHC score of ECA tumor cells was 0. (C2) Estrogen receptor (PR) IHC score of EMA tumor cells was 12. (D1) Vimentin IHC score of ECA tumor cells was 0. (D2) Vimentin of EMA tumor cells IHC score was12. Internal controls of stroma tissues showed strong positive result in both D1 and D2. (E1) Carcinoembryonic antigen (CEA) IHC score of ECA tumor cells was 12. (E2) Carcinoembryonic antigen (CEA) IHC score of EMA tumor cells was 2. (All were viewed at 400X magnification).

H&E and immunoreactivities for PR, ER, Vim and CEA can be seen in ECA and EMA. (Figure 1) The IHC findings are summarized in Table 2. Using a score of ≧ 4 points as a cutoff, all of the 4 markers showed a significant frequency difference between ECA and EMA tissue immunostainings. The PR-marker stained positive in 3 out of 14 (21.4%) ECA tumors with a median staining score of 0 and range of 0–9. The PR-marker stained positive in 14 out of 24 (58.3%) EMA tumors (p = 0. 027) with a median staining score of 5.00 and a range of 0–12 (p = 0.002). The ER-marker stained positive in 2 out of 14 (14.3%) ECA tumors with a median staining score of 0.50 and range of 0–6. The ER-marker stained positive in 18 out of 24 (75.0%) EMA tumors (p < 0.001) with a median staining score of 6.00 and a range of 0–12 (p < 0.001). The Vim-marker stained positive in 1 out of 14 (7.1%) ECA tumors with a median staining score of 0.00 and a range of 0–6. The Vim-marker stained positive in 16 out of 24 (66.7%) EMA tumors (p < 0.001) with a median staining score of 6.00 and a range of 0–12 (p < 0.001). The CEA-marker stained positive in 10 out of 14 (71.4%) ECA tumors with a median staining score of 5.00 and a range of 1–12. The CEA-marker stained positive in 4 out of 24 (16.7%) EMA tumors (p = 0.001), with a median staining score of 2.00 and a range of 0–9 (p < 0.001). These results found in Taiwanese women correspond with previous reports on Caucasian women[4,5,7-12].

**Table 2:** Immunohistochemical staining results (N: sample size)

		ECA (N = 14)	EMA (N = 24)	p-value
PR	Score 0–3	11 (78.6%)	10 (41.7%)	.027^a^
	Score 4–12	3 (21.4%)	14 (58.3%)	
	Median (Range)	0.00 (0–9)	5.00 (0–12)	.002^b^
ER	Score 0–3	12 (85.7%)	6 (25.0%)	< 0.001^a^
	Score 4–12	2 (14.3%)	18 (75.0%)	
	Median (Range)	0.50 (0–6)	6.00 (0–12)	< 0.001^b^
Vim	Score 0–3	13 (92.9%)	8 (33.3%)	< 0.001^a^
	Score 4–12	1 (7.1%)	16 (66.7%)	
	Median (Range)	0.00 (0–6)	6.00 (0–12)	< 0.001^b^
CEA	Score 0–3	4 (28.6%)	20 (83.3%)	.001^a^
	Score 4–12	10 (71.4%)	4 (16.7%)	
	Median (Range)	5.00 (1–12)	2.00 (0–9)	< 0.001^b^

1. Note: the ^a ^is chi-square test with continuity correction or Fisher's exact test, the ^b ^is the Mann-Whitney U test using exact significance

2. Using a score of ≧ 4 points as a cutoff, the immunostains are defined as "negative" for scores from 0 to 3, and "positive" for scores from 4 to 12 points.

We know that a typical EC-type immunoprofile staining pattern consists of PR-/ER-/Vim-/CEA+, and a typical EM-type immunoprofile staining pattern consists of PR+/ER+/Vim+/CEA-. In addition to the typical expression patterns of the ECA and EMA immunoprofiles, there are several other non-typical expression patterns for this 4-marker immunoprofile, as shown in Tables 3 and 4. When using the conventional 3-marker panel as a tool for the diagnostic distinction between ECA and EMA, there were 8 typical EC-type (ER-/Vim-/CEA+) expressions in the 14 primary ECA (57.14%), (Table 3) and 11 typical EM-type (ER+/Vim+/CEA-) expressions in the 24 primary EMA (45.83%). (Table 4) However, after adding the PR-marker to the traditional 3-marker (ER/Vim/CEA) panel, there still remained the same 8 typical EC-type (PR-/ER-/Vim-/CEA+) expressions in the 14 primary ECA (57.14%), (Table 3) and 9 typical EM-type (PR+/ER+/Vim+/CEA-) expressions in the 24 primary EMA (37.50%). (Table 4)

**Table 3:** Panel performance for the diagnosis of ECA (sample size 14)

	ER > 3	Vim > 3	CEA > 3	PR > 3	number	% with this panel
	-	-	-	-	2	14.29%
	-	-	-	+	1	7.14%
*	**-**	**-**	**+**	**-**	***8***	***57.14%***
	-	+	+	+	1	7.14%
	+	-	-	+	1	7.14%
	+	-	+	-	1	7.14%

1. -: score negative, +: score positive

2. Despite PR, panel expression with ER-/Vim-/CEA+ in mark-* was defined as a typical EC-type immunoprofile. Others were defined as non-typical EC-type immunoprofiles.

3. All 8 cases with typical EC-type (*) immunoprofile expression also exhibited PR (-).

**Table 4:** Panel performance for the diagnosis of EMA (sample size 24)

	ER > 3	Vim > 3	CEA > 3	PR > 3	number	% with this panel
	-	-	-	-	2	8.33%
	-	-	+	-	1	4.17%
	-	+	-	-	3	12.50%
	+	-	-	-	2	8.33%
	+	-	-	+	2	8.33%
	+	-	+	+	1	4.17%
*	**+**	**+**	**-**	**-**	***2***	***8.33%***
*	**+**	**+**	**-**	**+**	***9***	***37.50%***
	+	+	+	+	2	8.33%

1. -: score negative, +: score positive

2. Despite PR, panel expressions with ER+/Vim+/CEA- in mark-* were defined as typical EM-type immunoprofiles. Others were defined as non-typical EM-type immunoprofiles.

3. 11 cases with typical EM-type (*) immunoprofile expression also exhibited PR (+) in 9 cases, and PR (-) in 2 cases.

Table 5 presents the comparisons of the test effectiveness and performance between the 3-marker (ER/Vim/CEA) and 4-marker (PR/ER/Vim/CEA) panels with typical EC-type or typical EM-type in diagnostically distinguishing between 14 ECA and 24 EMA.

**Table 5:** Comparisons of the test effectiveness and performance between the 3-marker and 4-marker panels with typical EC type, typical EM type, typical EC type + PR (-) and typical EM type + PR (+) expressions in 14 ECA and 24 EMA

	ECA		EMA	
Panel compositions	3-marker Typical EC type	4-marker Typical EC type + PR(-)	3-marker Typical EM type	4-marker Typical EM type + PR(+)

Sensitivity (95% CI)	0.571 (0.414, 0.729)	0.571 (0.414, 0.729)	0.458 (0.296, 0.621)	0.375 (0.217, 0.533)
Specificity (95% CI)	0.958 (0.895, 1.000)	0.958 (0.895, 1.000)	1.000 (1.000, 1.000)	1.000 (1.000, 1.000)
Positive predictive value (95% CI)	0.889 (0.789, 0.989)	0.889 (0.789, 0.989)	1.000 (1.000, 1.000)	1.000 (1.000, 1.000)
Negative predictive value (95% CI)	0.793 (0.664, 0.922)	0.793 (0.664, 0.922)	0.519 (0.355, 0.682)	0.483 (0.320, 0.646)
Accuracy (95% CI)	0.80 (0.667, 0.933)	0.80 (0.667, 0.933)	0.686 (0.534, 0.837)	0.657 (0.502, 0.812)

1. -: negative score, +: positive score, 95% CI: 95% confidence interval.

2. Typical EC-type: immunoprofile expression with ER-/Vim-/CEA+

3. Typical EM-type: immunoprofile expression with ER+/Vim+/CEA-

4. PR (-) attributable to typical EC-type panel and PR (+) attributable to typical EM type panel.

## Discussion

Distinguishing between primary ECA and EMA before deciding the patient treatment plan is clinically important. Diagnostic difficulties may occur with some hysterectomy specimens when the tumor involves both the uterine endometrium and the endocervix. During preoperative assessment, there have been difficulties determining the primary tumor site using H&E alone. This study not only investigated the expression of 4 individual markers (PR, ER, Vim and CEA), but also evaluated whether panel performance and test effectiveness benefit from adding the PR-marker to the conventional 3-marker (ER/Vim/CEA) panel using IHC on a TMA techniques. This data will help in the referral and management of ECA and EMA cases worldwide.

There were some times when we inspected the surgical specimens during (or after) the staging procedure and found that the tumors involved both the endometrium and the endocervix. On other occasions, we obtained specimens from the biopsy of both sites of the lesions preoperatively or by fractional dilation and curettage (D&C), consisting of endocervical and endometrial curettage, which is already used as a classic standard procedure to diagnostically distinguish between EMA with (or without) cervical involvement and ECA with (or without) endometrial involvement. If the gross examination of hysterectomy specimens reveals indistinguishable primaries or the H&E histomorphology at these two adjacent lesions is similar, IHC with the traditional 3-marker (ER/Vim/CEA) or 4-marker (PR/ER/Vim/CEA) panel can be of great assistance in making the diagnostic distinction between ECA and EMA. In the 3-marker panel (ER/Vim/CEA), ECA tend to be ER-/Vim-/CEA+ and EMA tend to be ER+/Vim+/CEA-. Moreover, in the 4-marker panel (PR/ER/Vim/CEA), ECA tend to be PR-/ER-/Vim-/CEA+ and EMA tend to be PR+/ER+/Vim+/CEA-[4,5,7-12]. Within a tumor immunoprofile of either ECA or EMA, unexpected "aberrant" expressions may occur with any one or more of the 3- or 4-marker panels discussed. In this study, the IHC results of all the 4 individual markers (PR, ER, Vim and CEA), as well as their respective 3-marker (ER/Vim/CEA) or 4-marker (PR/ER/Vim/CEA) panels, showed significant differences in expression frequencies between the two types of adenocarcinomas (ECA *vs*. EMA).

We used the immunohistochemistry (IHC) on a tissue microarray (TMA) technique to investigate multiple specimens simultaneously in this extension study. This approach results in a dramatic reduction of time and cost compared with conventional histopathologic research techniques. TMA has become a popular tool for tissue-based research because it allows for the massive acceleration of studies correlating molecular in situ findings with clinico-pathological information. TMA has become specifically useful in surveys of tumor populations where it can be utilized to analyze the functions of specific markers or panels in neoplastic human tissues in both a comprehensive and efficient manner. On the other hand, the limitation of TMAs seems to be the insufficiency to demonstrate heterogeneity of the tumor because of the small size of tissue used; however, sampling with optimal cores in TMA was enough to show accuracies compared with whole mount sections. Studies have shown that increasing the number of cores results in only a slightly higher rate of validity and the noteworthy shortcomings of extra labor for constructing arrays, sample interpretation and data processing[22,23]. All 38 specimens were validated in this study.

IHC is no longer a qualitative immunoassay used only in research, but it is being increasingly employed as a semiquantitative or quantitative mode for the assessment of the presence of some therapeutic and prognostic biomarkers. Despite lacking a clear definition, many published papers have referred to the German Immunohistochemical Scoring System, as well as the term "semiquantative" to depict the scoring system for IHC interpretation. In fact, the "semiquantitative" scoring mechanism has generally been defined as the final immunoreactive score (IRS) is equal to the percentage of the extent of "quantitative" positive areas multiplied by the "qualitative" average of staining intensity. This "semiquantitative" scoring method has been reproduced and widely used in many laboratories[13,16,24-28].

There are a variety of IHC scoring methods including computer-based plans presented in literature, and there still seems to be no generally accepted protocol in research laboratories and clinical practices for rating and scoring immunostaining results. Comparing commercially derived computer-based programs with the conventional analyses by pathologists, there is still a lack of optimized and standardized IHC scoring algorithms. As a result, the objective accuracy did not significantly improve the clinical outcome measures. This study uses a widely accepted semi-quantitative scoring system for interpretation[13,16-20,24-28].

In other studies, it has been reported that IHC stained positive for the PR-marker in 89–96% of EMA, in contrast to 4–21% of ECA. IHC stained positive for the ER-marker in 67–97% of EMA, in contrast to 4–20% of ECA. IHC stained positive for the Vim-marker in 62–93% of EMA, in contrast to 7–14% of ECA; and IHC stained positive for the CEA-marker in 14–27% of EMA, in contrast to 62–93% of ECA.[4,5,7-12] In addition, we have already reported that when using the 3-marker (ER/Vim/CEA) panel, IHC stained positive for the ER-marker in 75.0% of EMA, in contrast to 14.3% of ECA. IHC stained positive for the Vim-marker in 66.7% of EMA, in contrast to 7.1% of ECA; and IHC stained positive for the CEA-marker in 16.7% of EMA, in contrast to 71.4% of ECA in our previous study[4,5]. In this study, we also found that IHC stained positive for the PR-marker in 58.3% of EMA, in contrast to 21.4% of ECA. The IHC results of these 4 individual markers and their respective 4-marker panel (PR/ER/Vim/CEA) revealed a significant diagnostic distinction between these 2 gynecologic malignancies (ECA *vs*. EMA) in this series of 14 cases of ECA and 24 cases of EMA.

McCluggage, et al. (2002) has already proposed that the conventional IHC 3-marker panel (ER, Vim and CEA) generally allows for a confident preoperative distinction between a primary endometrial and endocervical adenocarcinoma[3]. In this study, our purpose was to assess whether the 4-marker (PR/ER/Vim/CEA) panel containing PR could provide a more favorable performance than the conventional 3-marker (ER/Vim/CEA) panel in distinguishing between primary ECA and EMA.

In accordance with previous reports, we defined the typical EC-type immunoprofile as ER-/Vim-/CEA+, and the typical EM-type immunoprofile as ER+/Vim+/CEA-, using the conventional 3-marker (ER/Vim/CEA) panel. We also defined the typical EC-type immunoprofile as PR-/ER-/Vim-/CEA+, and the typical EM-type immunoprofile as PR+/ER+/Vim+/CEA-, using the 4-marker (PR/ER/Vim/CEA) panel[4,5,7-12].

In this study, with regard to the typical immunoprofiles in the conventional 3-marker (ER/Vim/CEA) panel, the sensitivity and negative predictive value (NPV) were 57.1% and 79.3% in ECA and 45.8% and 51.9% in EMA. However, considering the 4-marker (PR/ER/Vim/CEA) panel, the sensitivity and the negative predictive value (NPV) remained at 57.1% and 79.3% in ECA, but decreased to 37.5% and 48.3% in EMA with the typical EM-type immunoprofile. All these results indicated a high false-negative rate. If the staining results do not match up with the typical EC-type or EM-type immunoprofile, despite the PR-staining result, there is a high probability that the corresponding ECA or EMA tumor diagnosis will be doubtful and inconclusive. The low sensitivity and NPV of both panels are the limiting factors for the application of either the 3 or 4-marker immunoprofiles as a screening tool for ECA and EMA.

Moreover, regarding the 3-marker (ER/Vim/CEA) panel, the specificity and positive predictive value (PPV) were 95.8% and 88.9% in ECA and 100% and 100% in EMA. However, considering the 4-marker panel (PR/ER/Vim/CEA), the specificity and positive predictive value (PPV) remained the same at 95.8% and 88.9% in ECA and 100% and 100% in EMA. All these results indicated a low false-positive rate. If the staining result matches up with the typical EC-type or EM-type immunoprofile, despite the PR staining result, there is a high probability that the corresponding ECA or EMA tumor diagnosis will be accurate and conclusive. The high specificity and PPV of both panels are the promising factors for the application of either the 3 or 4-marker immunoprofiles as a confirmatory tool for the diagnosis of ECA and EMA.

Above all, accuracy means the degree of veracity. When using both 3-marker and 4-marker panels with typical EC-type (ER-/Vim-/CEA+ or PR-/ER-/Vim-/CEA+) immunoprofiles in definitively diagnosing primary ECA, both panels had the same accuracy rate (80.0% vs. 80.0%). On the other hand, when using both 3-marker and 4-marker panels with typical EM-type (ER+/Vim+/CEA- or PR+/ER+/Vim+/CEA-) immunoprofiles in definitively diagnosing primary EMA, the 3-marker panel had only a slightly higher accuracy rate compared with the 4-marker panel (68.6% vs. 65.7%).

It has been reported that most endocervical adenocarcinomas (ECAs) contain high-risk human papillomavirus (HPV) DNA, whereas endometrial adenocarcinomas (EMAs) rarely do. However, HPV data was not included in this report because we have already shown that the expression of p16^INK4a ^protein is significantly higher in endocervical adenocarcinomas than in endometrial adenocarcinomas, and the p16^INK4a ^marker seems to be a sensitive surrogate for the presence of HPV infection[29]. In this study, we did not include the p16^INK4a^-marker as an essential constituent in the panel along with ER/PR/Vim/CEA because our previous report demonstrated that adding the p16^INK4a ^to the conventional 3-marker (ER/Vim/CEA) or 4-marker (PR/ER/Vim/CEA) panel did not improve the panel performances in the diagnostic discrimination between ECA and EMA using the same IHC on a TMA techniques[4,5]. In addition, the interpretation of IHC data with p16^INK4a ^staining results is complicated because of the unclear biological significance of cytoplasmic staining and the lack of a universally accepted algorithm in the scoring methodology[30,31]. Some researchers regard cytoplasmic reactivity as an unexpected, unspecific event and consider nucleic p16^INK4a ^labeling in tumor cells to be only positive[16,32-35]. Others have stated, on the contrary, that both nucleic and cytoplasmic immunoreactivities in tumor cells are characteristic and are indeed due to p16^INK4a ^expression [36-40]. The knowledge about the functional meaning of cytoplasmic p16^INK4a ^expression is still limited and further large-scale studies are needed on various human tissues and tumors.

In summary, it is known that each of the four monoclonal antibodies directed against PR, ER, Vim, and CEA as well as a pre-existing panel, comprised of either 3 members (ER, Vim, CEA) or 4 members (PR, ER, Vim, CEA), could help in distinguishing between adenocarcinomas of endocervical origin and adenocarcinomas of endometrial origin. This study has demonstrated similar results to other studies in that a typical EC-type immunoprofile tends to be ER-/Vim-/CEA+ in ECA, whereas a typical EM-type immunoprofile tends to be ER+/Vim+/CEA- in EMA. The PR-marker tends to show negative expression in ECA, but positive expression in EMA. After assessing the panel performance and test effectiveness for sensitivity, specificity, NPV, PPV and accuracy of both 3- and 4-marker panels, we found that there was no supplemental benefit of adding the PR-marker to the conventional 3-marker (ER/Vim/CEA) panel in distinguishing between ECA and EMA. Despite the limited number of cases, the data provides significant and valuable references for further investigation to determine other useful immunoprofiles or panels that will definitively distinguish between ECA and EMA.

## Conclusion

Distinguishing between ECA and EMA can often be accomplished by routine gross and histological examinations, but some cases reveal tumors with undetermined origins or overlapping histomorphologic features. An accurate diagnosis may require the use of ancillary IHC stains. Data from this TMA study provides valuable references of consistency between Taiwanese and Caucasian women. We found that the 3-marker (ER/Vim/CEA) panel can sufficiently provide a more advantageous, cost-effective, and easier means for appropriately distinguishing between ECA and EMA. Although the 4-marker (PR/ER/Vim/CEA) panel also reveals statistically significant results, it is a waste of resources. Based on our data, we found that adding the PR-marker offers no supplemental benefit to the pre-existing 3-marker (ER/Vim/CEA) panel in the diagnostic distinction between ECA and EMA. Therefore, we still recommend the conventional 3-marker panel with ER, Vim and CEA as sufficient, appropriate and useful in distinguishing the difference in origin between ECA and EMA.

## List of abbreviations used

ECAs: Endocervical adenocarcinomas; EMAs: Endometrial adenocarcinomas; p16: p16^INK4a^; IHC: Immunohistochemistry; TMA: tissue microarray; ER: Estrogen receptor; PR: Progesterone receptor; Vim: Vimentin; CEA: Carcinoembryonic antigen; H&E: Hematoxylin and eosin; D&C: dilation and curettage; NPV: Negative predictive value; PPV: Positive predictive value.

## Competing interests

The authors declare that they have no competing interests.

## Authors' contributions

CPH, LFK performed experiments and wrote the manuscript. MYL performed the statistical analysis. KCC, CLL, PHW, YST, CLK participated in its design and coordination. TSW edited the draft manuscript. All authors read and approved the final manuscript.
